# Prediction of moderate and severe toxicities of chemotherapy in older patients with cancer: a propensity weighted analysis of ELCAPA cohort

**DOI:** 10.1093/oncolo/oyae157

**Published:** 2024-07-06

**Authors:** Marc-Antoine Benderra, Elena Paillaud, Amaury Broussier, Richard Layese, Claudia M Tapia, Soraya Mebarki, Pascale Boudou-Rouquette, Marie Laurent, Monica Piero, Florence Rollot-Trad, Joseph Gligorov, Philippe Caillet, Florence Canoui-Poitrïne

**Affiliations:** Université Paris-Est Créteil, INSERM, IMRB, F-94010 Créteil, France; AP-HP, Hopital Henri-Mondor, Public Health Department and Clinical Research Unit (URC Mondor), F-94010 Créteil, France; Institut Universitaire de Cancérologie (IUC), AP-HP, Sorbonne Université, F-75013 Paris, France; Université Paris-Est Créteil, INSERM, IMRB, F-94010 Créteil, France; AP-HP, Hopital Européen Georges Pompidou, Paris Cancer Institute CARPEM, Department of Geriatrics, F-75015 Paris, France; Université Paris-Est Créteil, INSERM, IMRB, F-94010 Créteil, France; AP-HP, Hopitaux Henri Mondor/Emile Roux, Department of Geriatrics, F-94456 Limeil-Brevannes, France; Université Paris-Est Créteil, INSERM, IMRB, F-94010 Créteil, France; AP-HP, Hopital Henri-Mondor, Public Health Department and Clinical Research Unit (URC Mondor), F-94010 Créteil, France; Université Paris-Est Créteil, INSERM, IMRB, F-94010 Créteil, France; Université Paris-Est Créteil, INSERM, IMRB, F-94010 Créteil, France; AP-HP, Hopital Européen Georges Pompidou, Paris Cancer Institute CARPEM, Department of Geriatrics, F-75015 Paris, France; AP-HP, Hopital Cochin, Cancer Research for PErsonalized Medicine (CARPEM), Department of Medical Oncology, ARIANE Program, Paris Cité University, F-75015 Paris, France; Université Paris-Est Créteil, INSERM, IMRB, F-94010 Créteil, France; AP-HP, Hopitaux Henri Mondor/Emile Roux, Department of Geriatrics, F-94456 Limeil-Brevannes, France; AP-HP, Hopital Cochin, Cancer Research for PErsonalized Medicine (CARPEM), Department of Medical Oncology, ARIANE Program, Paris Cité University, F-75015 Paris, France; Hopital Institut Curie, Unité d'oncogériatrie, Department of Supportive Care, F-92210 Saint-Cloud, France; Hopital Institut Curie, Unité d'oncogériatrie, Department of Supportive Care, F-92210 Saint-Cloud, France; Institut Universitaire de Cancérologie (IUC), AP-HP, Sorbonne Université, F-75013 Paris, France; AP-HP, Hopital Tenon, Department of Medical Oncology, F-75020 Paris, France; Université Paris-Est Créteil, INSERM, IMRB, F-94010 Créteil, France; AP-HP, Hopital Européen Georges Pompidou, Paris Cancer Institute CARPEM, Department of Geriatrics, F-75015 Paris, France; Université Paris-Est Créteil, INSERM, IMRB, F-94010 Créteil, France; AP-HP, Hopital Henri-Mondor, Public Health Department and Clinical Research Unit (URC Mondor), F-94010 Créteil, France

**Keywords:** cancer, older patients, geriatric assessment, chemotherapy toxicities

## Abstract

**Background:**

Currently available predictive models for chemotherapy-related toxicity are not sufficiently discriminative in older patients with cancer and do not consider moderate toxicities. The purpose of this study was to identify factors associated with moderate and severe chemotherapy toxicities in older patients with cancer.

**Materials and methods:**

Patients aged 70+ recruited in the prospective ELCAPA cohort were analyzed. A total of 837 patients with data on toxicities had received chemotherapy without other systemic treatment and were included between 2015 and 2022. To adjust for any imbalances in the distribution of covariates between patients receiving single-agent chemotherapy vs combination chemotherapy, we applied overlap weighting (a propensity-score-based technique). We used multinomial logistic regression.

**Results:**

Median (interquartile range) age was 81 (77-84). Forty-one percent experienced moderate toxicity, and 33% experienced severe toxicity. Hematologic toxicities accounted for 53% of severe toxicities and 66% of moderate toxicities. Age <80 years, cancer type, metastatic status, Eastern Cooperative Oncology Group performance status (ECOG-PS) >1, no cognitive impairment were associated with combination chemotherapy decision. In a univariate analysis with overlap weighting, no factors were associated with moderate toxicity. Hemoglobin < 10 g/dL and a CIRS-G score >12 were associated with severe toxicity. In a multivariate analysis, only hemoglobin < 10 g/dL was independently associated with severe toxicity, adjusted OR 2.96 (95% CI, 1.20-7.29).

**Conclusion:**

By addressing indication bias for combination chemotherapy decision, only anemia and not cancer type, combination chemotherapy was predicting for severe chemotherapy-related toxicity in older patients with cancer. We did not find any predictors of moderate chemotherapy-related toxicity.

## Teaser

Predictive models for chemotherapy-related toxicity are not sufficiently discriminative in older patients with cancer and do not consider moderate toxicities. This study identified factors associated with moderate and severe chemotherapy toxicities in older patients with cancer.

**Implications for practice**

Moderate chemotherapy toxicities are common among older patients with cancer. They can result in treatment discontinuation, alterations in treatment plans, loss of independence, and diminished quality of life. However, these moderate toxicities are not taken into account in chemotherapy toxicity prediction scores. In our study, we observed moderate toxicities in 41% of patients and severe toxicities in 33% of patients. We did not find any correlations between moderate chemotherapy-related toxicities and geriatric assessment components, comorbidities, or cancer-related variables. Nevertheless, we discovered a significant association between severe toxicity and anemia.

## Introduction

The International Society of Geriatric Oncology defines older cancer patients as those aged over 70.^1^ In France, where 1 in every 2 cancers is diagnosed in an individual aged 70 or over,^2^ it is essential to provide optimal treatment for these often frail patients.^3,4^ Even though the benefits of chemotherapy (in terms of overall survival) are similar in younger and older patients,^5,6^ the increased incidence of chemotherapy-related toxicities with age^7,8^ tends to discourage physicians from prescribing this treatment modality to the latter age group.^9^

Efforts to refine the risk-benefit assessment of chemotherapy and avoid undertreatment have led to the development of algorithms that predict toxicity in older cancer patients, such as the Chemotherapy Risk Assessment Scale for High-Age Patients (CRASH)^10^ and the Cancer and Ageing Research Group (CARG) scale.^11^ Several external evaluations of both the CRASH and CARG scores have been published,^12,13^ revealing conflicting results. These tools exhibit only moderate predictive capabilities and have not been widely adopted in clinical practice. The CRASH takes 20-30 minutes to complete and so is less favored than the faster-to-complete CARG score, which gave an area under the curve of 0.72 for predicting grade 3-5 chemotherapy toxicities.^11^ Unfortunately, the currently available algorithms do not consider moderate toxicities or evaluate comorbidities comprehensively. The inclusion of moderate toxicities is crucial for older patients, given the potential impact on autonomy, quality of life, and treatment adherence.^14,15^ Moreover, the algorithms are subject to selection bias because the clinical factors that influence treatment decisions (such as overall health, frailty, and comorbidities) have not been incorporated. Consequently, the treatment indication is a confounding factor in these algorithms.^16^ Given that comorbidities are more prevalent among older patients^10^ and are linked to low chemotherapy completion rates in patients with colon,^17,18^ breast^19^ and lung cancers,^15^ the accurate quantification of comorbid conditions is crucial. The Cumulative Illness Rating Scale for Geriatrics (CIRS-G) is a comprehensive prognostic tool tailored for older patients^20,21^ but is underused; the faster-to-complete Charlson score^22^ is often preferred.

The primary objective of the present study was to identify variables associated with the occurrence of moderate and severe toxicities in a cohort of older cancer patients. The secondary objective was to distinguish between risk factors for toxicity and risk factors related to the treatment indication.

## Methods

### Design and patients

We analyzed data from the Elderly Cancer Patients (ELCAPA) prospective, multicentre, open-cohort study (NCT02884375). Nineteen investigating centers in the Paris urban area of France recruited consecutive patients (1) aged over 70, (2) newly diagnosed with a solid tumor or hematological cancer, and (3) referred for a geriatric assessment (GA) prior to choice of cancer treatment. Verbal, informed consent was obtained from all study patients prior to inclusion. The study protocol was approved by the appropriate independent ethics committee (*CPP Ile-de-France I*, Paris, France; reference: IORG0009918). For the present analysis, we selected ELCAPA cohort patients had received chemotherapy (without any other systemic treatment, that is, targeted therapy or immunotherapy) between 2015 and 2022 (*n* = 1172). The oncologist responsible for prescribing chemotherapy was informed of the geriatric assessment results.

### Data collection

Baseline data were collected prospectively at the time of the initial GA. The GA was performed by a senior geriatrician with expertise in oncology. The variables considered in the GA were age, sex, inpatient vs outpatient status at the time of inclusion, Eastern Cooperative Oncology Group performance status (ECOG-PS), tumor site, metastatic status (yes/no), body mass index (BMI), the Mini Nutritional Assessment (MNA) score ≥10% weight loss in the previous 6 months (yes/no), a “timed up-and-go” (TUG) test completion time >20 seconds (yes/no), a fall in the previous 6 months (yes/no), the Mini-Mental State Examination score, the Activities of Daily Living (ADL) score,^13^ the Instrumental Activities of Daily Living (IADL) score,^14^ the family environment (marital status, and the presence of a family caregiver or not), and the number of prescription medications taken daily. The CIRS-G score was used to measure the comorbidity burden at the time of the baseline CGA. The CIRS-G rates comorbidities in 14 organ systems on a 5-point scale ranging from 0 (no dysfunction) to 4 (extremely severe dysfunction); this gives a total score ranging from 0 (best) to 56 (worst). Severe comorbidity was defined by a score of 3 or 4 for each organ system concerned. The CIRS-G score was analyzed according to the following approaches: total score, number of organ systems with a score ≥1, and organ systems with a score ≥3. A score ≥3 defined severe comorbidities. Other comorbidities were also recorded: ischemic cardiopathy, heart failure, arrhythmia, hypertension, diabetes mellitus, obesity (BMI > 30 kg/m^2^), chronic obstructive pulmonary disease, renal failure (Cockcroft creatinine clearance rate < 60 mL/minute), severe renal failure (Cockcroft creatinine clearance rate < 30 mL/minute), liver failure, depression, and cognitive impairment. The presence or absence of depressive syndrome and/or cognitive impairment was judged by the ELCAPA study investigators.

### Outcome

The patient was monitored throughout the course of chemotherapy or (if the latter was not completed) for up to 6 months. The primary endpoint was toxicity during chemotherapy. Toxicities were recorded for each course of chemotherapy and graded according to the National Cancer Institute Common Terminology Criteria for Adverse Events (NCI CTCAE, version 5.0) as moderate (grade 2) or severe (grade 3 or 4).

### Statistical analyses

Descriptive analyses of the patient characteristics, tumor characteristics and GA results were performed. The incidences of specific toxicity categories (hematologic and non-hematologic) and CTCAE grades (0-1, 2, or 3-4) were calculated. Multinomial logistic regression was used to examine the association between grade 2 and grade 3-4 toxicity and the following variables: age, sex, BMI, ECOG-PS, cancer type (gastrointestinal (GI) (reference), gynecological, genitourinary (GU), lung, or other), prechemotherapy laboratory test results (leukocyte, neutrophil, lymphocyte, and platelet counts, hemoglobin level, liver function tests, albumin, creatinine clearance rate (calculated using the Cockroft-Gault formula), the neutrophil–lymphocyte ratio, and the platelet–lymphocyte ratio), and GA variables (≥10% weight loss in the previous 6 months, a “timed up-and-go” (TUG) test” completion time >20 seconds, a fall in the previous 6 months, the ADL score,^9^ the Instrumental Activities of Daily Living (IADL) score,^23^ the family environment (marital status and the presence of a family caregiver or not), cognitive disorders, visual or hearing disorder, and the number of prescription medications taken daily).

For continuous variables, the Youden Index was used to identify the cutoff with the highest sensitivity and specificity for classifying the presence or absence of toxicity. Variables with *P* < .2 and clinically relevant variables were fed into a multivariate, multinomial logistic regression model.

To adjust comparisons of patients having received single-agent chemotherapy vs combination chemotherapy, we applied a propensity score (PS) with the following components: age (as a continuous variable), cancer type, CIRS-G score, ECOG-PS, hemoglobin < 10 g/dL, albuminemia < 35 g/l, and severe renal failure (creatinine clearance rate <30 mL/minute) (Supplementary Table S1). Missing data were imputed using classification and regression trees, using the *MICE* package in R. In overlap weighting (OW), each patient was assigned with a weight proportional to the probability of belonging to the opposite group.^24^ Non-imputed data were used in the sensitivity analysis. In a sensitivity analysis, we used multinomial logistic regression to assess hematological and non-hematological toxicities separately.

The threshold for statistical significance was set to *P* < .05. All tests were 2-tailed, and statistical analyses were performed with R software (version 4.0.3).^25^

## Results

### Patients

Of the 1172 patients included in the ELCAPA cohort between 2015 and 2022 with available data on toxicities, 837 had received chemotherapy without other systemic treatment and were included in our analysis.

### Clinical characteristics

The median [interquartile range (IQR)] age was 81 (77-84), 491 (59%) of the patients were women, and 484 (58%) had metastatic cancer (Tables 1 and 2). Of the 837 patients included, 265 (32%) had GI cancer, 265 (32%) had gynecologic cancer, including 159 (60%) with breast cancer, 110 (13%) had a GU cancer, 82 (10%) had lung cancer, and 114 (13%) had another type of cancer. Combination chemotherapy was administered to 512 patients (64%). According to the results of the GA, 42% of patients lived alone. The mean ± SD CIRS-G score was 10.0 ± 4.64. The ADL and IADL were impaired in 13% and 39% of patients, respectively. Mobility (as evaluated in the TUG test) was impaired in 17% of the patients. Cognitive impairment was detected in 17% of the patients, and depressive syndrome was detected in 18%.

**Table 1.** Demographic and clinical characteristics of the study participants.

Characteristic	No. of patients (*n* = 837)	% Patients
Median age (range)		
70-79	359	43
80-89	442	53
90-99	36	4
Sex		
Male	346	41
Female	491	59
ECOG-PS		
0	138	16
1	368	44
2	242	29
3	72	9
4	10	1
Missing	7	1
Cancer type		
Digestive	265	32
Gynaecological	265	32
GU	110	13
Lung	82	10
Other	114	13
Missing	1	0
Metastatic status		
Metastatic	484	58
Non-metastatic	352	42
Missing	1	0
Regimen		
Single-agent chemotherapy	293	32
Combination chemotherapy	512	64
Missing	32	4
BMI, kg/m^2^		
<22	235	28
22-25	238	29
≥25	352	42
Missing	12	1
Hemoglobin, g/dL		
< 10	129	15
≥10	680	81
Missing	28	4
Platelet count, ×10^3^/μL		
< 150	67	8
≥150	742	89
Missing	28	3
Creatinine clearance rate, mL/minute		
<30	37	4
30-60	410	49
≥60	337	40
Missing	53	6
Albumin, g/dL		
<35	265	32
≥35	433	52
Missing	139	16

Abbreviations: BMI, body mass index; ECOG-PS, Eastern Cooperative Oncology Group Performance Status; GU, genito-urinary.

**Table 2.** The results of the baseline GA.

Characteristic	No. of patients (*n* = 837)	% Patients
GA location		
GA during a consultation	661	79
GA upon hospital admission	176	21
IADL		
7-8	510	61
< 7	320	38
Missing	7	1
ADL		
6	727	87
≥5	106	13
Missing	4	0
Fall in the past 6 months		
No	656	78
Yes	168	20
Missing	13	2
CIRS-G score		
0-6	199	24
7-12	374	45
> 12	223	26
Missing	41	5
Concomitant drugs		
<5	290	35
≥5	501	60
Missing	46	5
Cognitive impairment^*^		
No	640	76
Yes	135	16
Missing	62	8
Depressive syndrome^*^		
No	664	79
Yes	141	17
Missing	32	4
TUG test time		
≤20 seconds	597	71
>20 seconds	118	14
Missing	122	15
Lives alone at home		
No	482	58
Yes	353	42
Missing	2	0

^*^As judged by the ELCAPA investigator.

Abbreviations: ADL, activities of daily living; CIRS-G, Cumulative Illness Rating Scale for Geriatrics; GA: geriatric assessment; IADL, Instrumental Activities of Daily Living; TUG, timed up-and-go

### Chemotherapy toxicities

**Figure 1.**
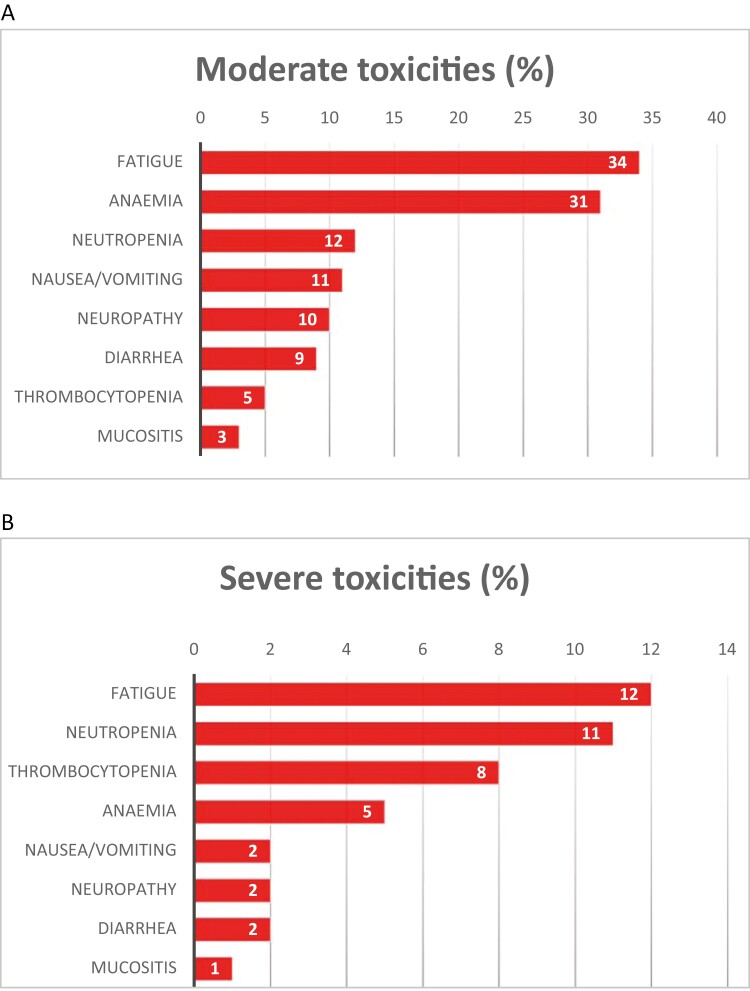
Incidences of moderate (A) and severe (B) chemotherapy-related toxicities.

At least one moderate or severe toxicity event was observed in 618 (74%) of the 837 patients analyzed. Severe toxicity was observed in 279 of these 618 patients (33%) (107 patients (30%) without metastatic cancer and 171 patients (35%) with metastatic cancer) (Figure 1). The most frequent severe toxicities were fatigue (12%), neutropenia (11%), thrombocytopenia (8%), anemia (5%), nausea/vomiting (2%), and diarrhea (2%). Moderate toxicities affected 339 patients (41%); the most frequent were fatigue (34%), anemia (31%), neutropenia (12%), and nausea/vomiting (11%). Peripheral neuropathy was a concern for 10% of patients and had a significant impact on their daily activities, according to the CTCAE. The proportion of patients with moderate toxicity was similar in individuals with non-metastatic cancer (41%) and those with metastatic cancer (40%). Hematologic toxicities were frequent and accounted for 53% of the severe toxicities and 66% of the moderate toxicities.

**Figure 2.**
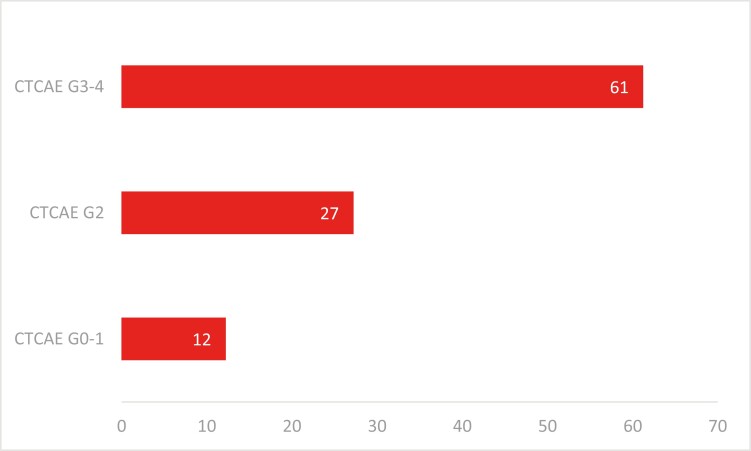
Description of percentage toxicity grades among patients who discontinued chemotherapy (*n* = 82).

Chemotherapy was discontinued as a result of toxicity in 82 (10%) of the 837 patients included in our analysis (severe toxicity in 50 (61%); moderate toxicity in 22 (27%); CTCAE grades 0-1 in 10 (12%) patients). (Figure 2).

Factors associated with combination chemotherapy were age <80 years, cancer type, metastatic status, ECOG-PS >1, and no cognitive impairment.

### Predictive variables associated with moderate and severe toxicities

In a univariate analysis, only combination chemotherapy was associated with moderate toxicity. Several variables were associated with the occurrence of severe toxicity: ECOG-PS > 1, hemoglobin < 10 g/dL, albuminemia < 35 g/L, a CIRS-G score >12, and an impaired TUG time. In contrast, female sex and a TUG time <20 s were associated with fewer severe toxicities.

In a multivariate analysis, lung cancer was associated with moderate toxicity, and hemoglobin<10 g/dL and GU cancer were associated with severe toxicity (Table 3). ECOG-PS, ADL, and IADL were correlated (correlation coefficient > 0.3). We chose to include only ECOG-PS in the multivariate analysis to avoid multicollinearity.

**Table 3.** Associations between patient characteristics and chemotherapy toxicities, before overlap weighting.

	Univariate analysis						Multivariate analysis					
	Moderate toxicities			Severe toxicities			Moderate toxicities		P	Severe toxicities		P
Variable&	OR	95%CI	*P*	OR	95%CI	*P*	OR	95%CI		OR	95%CI	
Female sex	0.82	0.58-1.16	.26	0.70	0.49-1.00	.05	0.99	0.53-1.83	.97	1.02	0.54-1.95	.94
>80 years of age	0.78	0.55-1.09	.15	1.04	0.72-1.49	.85	0.87	0.54-1.41	.57	1.17	0.70-1.94	.55
Metastatic status	1.09	0.77-1.54	.62	1.32	0.92-1.89	.13	0.93	0.57-1.53	.79	0.80	0.48-1.33	.38
ECOG-PS > 1	1.40	0.98-2.00	.07	1.70	1.17-2.46	.005	1.68	0.97-1.45	.39	1.13	0.64-2.00	.68
Polychemotherapy	1.43	1.00-2.04	.05	1.06	0.73-1.53	.76	1.32	0.80-2.19	.28	1.08	0.64-1.82	.76
Hemoglobin < 10 g/dl	1.72	0.98-3.02	.06	2.98	1.72-5.17	<.001	1.94	0.82-4.59	.13	2.76	1.19-6.42	.02
Creatine clearance rate < 30 mL/minute	1.30	0.48-3.52	.61	2.53	0.99-6.44	.05	0.82	0.20-3.27	.77	1.73	0.50-6.00	.38
Albuminemia < 35 g/L	1.16	0.78-1.72	.47	1.90	1.27-2.84	.002	0.92	0.53-1.59	.75	1.43	0.81-2.50	.21
6-month weight loss	1.41	0.92-2.16	.12	1.38	0.88-2.15	.16	1.16	0.66-2.05	.61	0.97	0.53-1.76	.92
CIRS-G > 12	1.40	0.93-2.11	.10	1.69	1.12-2.57	.01	1.07	0.60-1.93	.82	1.52	0.85-2.74	.16
ADL < 6	1.17	0.69-1.97	.56	1.19	0.69-2.05	.53						
IADL < 7	1.23	0.86-1.76	.25	1.40	0.97-2.03	.07						
TUG < 20 s	0.72	0.43-1.23	.23	0.59	0.34-1.00	.05	0.75	0.38-1.45	.39	0.90	0.45-1.82	.77
Cognitive impairment	0.93	0.57-1.51	.76	1.38	0.85-2.22	.19	0.64	0.33-1.21	.17	1.00	0.53-1.88	.99
BMI <22 vs 22-25 kg/m^2^ >25 vs 22-25 kg/m^2^	0.87 1.19	0.56-1.36 0.79-1.80	.55 .41	0.94 1.07	0.59-1.49 0.70-1.65	.41 .75						
Gynaecological cancer^*^	0.99	0.65-1.50	.95	0.75	0.48-1.17	.21	1.45	0.74-2.81	.28	1.39	0.69-2.78	.35
GU cancer^*^	1.26	0.69-2.32	.45	1.75	0.96-3.19	.07	1.22	0.51-2.89	.66	2.47	1.06-5.72	.04
Lung cancer^*^	1.68	0.86-3.29	.13	1.45	0.72-2.91	.30	3.10	1.13-8.52	.03	2.72	0.93-7.97	.07
Other cancer^*^	0.75	0.44-1.28	.13	0.65	0.37-1.14	.13	1.15	0.53-2.49	.72	0.92	0.41-2.08	.84

^&^For continuous variables, the Youden Index30 was used to identify the cutoff with the highest sensitivity and specificity for classifying the presence or absence of toxicity. Variables with *P* < .1 in the univariate analysis and clinically relevant variables (single-agent chemotherapy or combination chemotherapy) were examined further in a multivariate logistic regression model.

^*^Reference: digestive cancer.

Abbreviations: ADL, activities of daily living; BMI, body mass index; CIRS-G, Cumulative Illness Rating Scale for Geriatrics; ECOG-PS, Eastern Cooperative Oncology Group Performance Status; GU, genito-urinary; IADL, Instrumental Activities of Daily Living; TUG, timed up-and-go.

### Adjusted comparisons of patients having received a single-agent chemotherapy vs combination chemotherapy

All variables were well balanced between patients having received a single-agent chemotherapy vs combination chemotherapy after OW (Supplementary Table S2).

After OW, no variables were associated with moderate toxicity in univariate or multivariate analyses. In a univariate analysis after OW, 2 variables were associated with severe toxicity: hemoglobin < 10 g/dL and CIRS-G score > 12. In a multivariate logistic regression after OW, only hemoglobin < 10 g/dL was found to be independently associated with severe toxicity (Table 4).

**Table 4.** Associations between patient characteristics and chemotherapy toxicities, after overlap weighting

	Univariate analysis						Multivariate analysis					
	Moderate toxicities			Severe toxicities			Moderate toxicities		p	Severe toxicities		p
Variable^&^	OR	95%CI	*p*	OR	95%CI	*p*	OR	95%CI		OR	95%CI	
Female sex	0.88	0.51-1.49	.63	0.86	0.49-1.50	.59						
>80 years of age	0.79	0.46-1.35	.38	0.95	0.54-1.68	.87						
Metastatic status	0.91	0.54-1.55	.74	1.06	0.61-1.86	.83						
ECOG-PS > 1	1.37	0.80-2.35	.25	1.68	0.96-2.94	.07	1.12	0.62-2.01	.71	1.26	0.68-2.33	.46
Polychemotherapy	1.38	0.82-2.33	.23	1.14	0.66-1.98	.63						
Hemoglobin < 10 g/dl	1.82	0.75-4.38	.19	3.45	1.46-8.14	.005	1.78	0.71-4.47	.22	2.96	1.20-7.29	.02
Creatine clearance rate < 30 mL/minute	1.48	0.33-6.55	.49	2.80	0.68-11.56	.15	1.40	0.31-6.32	.66	2.42	0.56-10.33	.23
Albuminemia < 35 g/L	0.98	0.57-1.69	.94	1.49	0.85-2.61	.16	0.78	0.43-1.40	.40	1.03	0.56-1.89	.93
6-month weight loss	1.45	0.77-2.75	.25	1.43	0.74-2.77	.29						
CIRS-G > 12	1.58	0.86-2.90	.14	1.87	1.00-3.48	.05	1.51	0.80-2.85	.20	1.62	0.84-3.14	.15
ADL < 6	1.18	0.54-2.61	.68	1.32	0.59-2.97	.50						
IADL < 7	1.14	0.67-1.96	.63	1.39	0.80-2.43	.25						
TUG < 20 s	0.52	0.25-1.05	.07	0.56	0.26-1.16	.12	0.53	0.25-1.11	.09	0.65	0.30-1.42	.28
Cognitive impairment	0.89	0.45-1.76	.74	1.20	0.61-2.38	.59						
BMI <22 vs 22-25 kg/m^2^ >25 vs 22-25 kg/m^2^	1.12 1.34	0.57-2.22 0.72-2.49	.74 .36	1.27 1.17	0.62-2.56 0.61-2.26	.51 .63						
Gynaecological cancer^*^	0.87	0.45-1.68	.69	0.73	0.37-1.46	.38						
GU cancer^*^	1.14	0.46-2.82	.77	1.34	0.54-3.34	.52						
Lung cancer^*^	0.98	0.34-2.79	.97	0.84	0.28-2.55	.76						
Other cancer^*^	0.73	0.33-1.64	.45	0.67	0.29-1.55	.35						

^&^For continuous variables, the Youden Index30 was used to identify the cutoff with the highest sensitivity and specificity for classifying the presence or absence of toxicity. Variables with *P* < .1 in the univariate analysis and clinically relevant variables (single-agent chemotherapy or combination chemotherapy) were examined further in a multivariate logistic regression model.

^*^Reference: digestive cancer.

Abbreviations: ADL, Activities of Daily Living; BMI, body mass index; CIRS-G, Cumulative Illness Rating Scale for Geriatrics; ECOG-PS, Eastern Cooperative Oncology Group Performance Status; GU, genito-urinary; IADL, Instrumental Activities of Daily Living; TUG, timed up-and-go.

### Sensitivity analysis of hematologic and non-hematologic toxicities

The variables associated with hematological toxicities differed from those associated with non-hematological toxicities (Supplementary Tables S3 and S4). In a univariate analysis, male sex, ECOG-PS >1, hemoglobin < 10 g/dL, albuminemia < 35 g/L, and severe renal failure were associated with both moderate and severe hematological toxicities. For non-hematological toxicities, only combination chemotherapy was associated with moderate toxicity, and no variables were associated with severe toxicity. In a multivariate analysis, hemoglobin < 10 g/dL was associated with moderate and severe hematological toxicities. Gynecological cancer was associated with severe non-hematological toxicities.

## Discussion

Although older patients face a high risk of developing chemotherapy-related toxicities, the tools available for identifying at-risk individuals focus mainly on severe toxicities and neglect selection (indication) bias and the impact of moderate toxicities. On one hand, moderate toxicities can significantly impact the quality of life and may lead to dose reduction or delays in chemotherapy^14^; on the other, the choice of the optimal treatment for an older patient must notably take into account the type of cancer, comorbidities, and frailty—a cornerstone of geriatric oncology. To the best of our knowledge, the present study is the first large-scale investigation of predictors of moderate and severe chemotherapy toxicities in older adults with cancer while addressing selection bias. We did not find any associations between moderate chemotherapy-related toxicities and GA components, comorbidities, or cancer-related variables. However, we found that severe toxicity was significantly associated with anemia. Our results also indicate that cancer type reflects the treatment choice and is not a risk factor for chemotherapy-related toxicities.

Our study had several strengths. First, we meticulously took into account all our analyses for the decision-making process and the administration of single-agent chemotherapy vs combination chemotherapy by applying OW. This approach helped us to predict the likelihood of toxicity in a patient receiving combination chemotherapy based on the various factors associated with the indication of combination chemotherapy, irrespective of the actual treatment received. This approach mitigates the confounding effects of combination chemotherapy, which might otherwise have influenced the interpretation of variables associated with toxicity. In the field of oncology, treatment choices are often based on clinical expertise and indices of the patient’s functional abilities (eg, the Karnofsky Performance Status Scale). Consequently, combination chemotherapy might be recommended for patients in better general condition and at lower risk of severe toxicity. Conversely, monotherapy can be selected for patients unable to tolerate combination chemotherapy.

Moderate chemotherapy toxicities in older patients can lead to treatment discontinuation, changes in treatment, loss of autonomy, and decreased quality of life. We chose to group grade 0-1 toxicities because according to the CTCAE, they do not impact ADL. In contrast, grade 2 toxicities affect ADL and were considered to be moderate toxicities. Kalsi et al. found that low-grade toxicities accounted for 35% of dose modifications and 39.1% of early discontinuations of chemotherapy.^14^ In our study, 42% of the patients experienced at least one moderate toxicity—primarily fatigue and anemia. Peripheral neuropathy can prompt a dose reduction or temporary discontinuation of chemotherapy and was observed in 10% of the patients. This side effect is particularly concerning in older adults because it is not reversible and can increase the risk of falls. Diarrhea was also a relevant toxicity in older patients, 9% of whom experienced a grade 2 event. Although we did not have specific data on dose adjustments for these patients, grade 2 peripheral neuropathy typically leads to a dose reduction or temporary discontinuation of chemotherapy. However, our study might have underestimated the occurrence of moderate toxicities because the latter were assessed subjectively by oncologists in a real-life practice setting. This underestimation has also been observed in clinical trials, which focus on grade 3 or 4 toxicities. Patient-reported outcome measures might be of value in determining acceptable toxicity levels for patients, and so the use of these measures should be encouraged. Culakova et al^15^ applied the Patient-Reported Outcomes Common Terminology Criteria for Adverse Events and found that 86.1% of their patients reported moderate toxicities.

Even though 63% of our patients received combination chemotherapy, the incidence of severe toxicity (36%) was relatively low—notably when compared with the CARG and CRASH cohorts, where the corresponding values were over 50%.^10,11^ Feliu et al ^26^ reported a similar incidence of severe toxicity (33.5%). These inter-study disparities in the toxicity rate can be attributed to differences in study populations. In the CARG cohort, for example, 29% of patients had lung cancer, and 27% had digestive cancer. In the CRASH cohort, 20% of patients had lung cancer. In our study, the majority of the patients had digestive cancers (33%) or gynecological cancers (30%, including 60% of breast cancer), and only 11% had lung cancer. Furthermore, the relatively low incidence of severe toxicity observed in our study might be explained by the systematic administration of a GA in the ELCAPA cohort. As reported in the literature, the results of a GA can influence the treatment decisions made by oncologists. For example, Caillet et al reported that a GA led to treatment modifications in 21% of patients and that 80.8% of these modifications resulted in a reduction in treatment intensity.^27^ Furthermore, it has been shown that a geriatric assessment-driven intervention prior to the initiation of chemotherapy was associated with a lower incidence of chemotherapy toxicities.^28,29^ These observations highlight the importance of selecting chemotherapy carefully and monitoring adverse events closely.

In the present study, lung cancer was associated with severe toxicities, while genitourinary (GU) cancer was associated with moderate toxicities, as determined by multivariate analysis. Interestingly, these associations were not found after the data were weighted with a propensity score, which raises the question of whether specific tools or scores are of value for predicting chemotherapy toxicities for a given type of cancer. For instance, the CARG breast cancer score was developed to predict severe toxicities resulting from adjuvant chemotherapy in patients with breast cancer.^30^ However, a tool that could be applied to all cancer types, taking into account the chemotherapy intent (type, number of drugs) would probably be more readily adopted in routine clinical practice. Furthermore, the use of several scores might complicate clinical practice while providing uncertain benefits.

Our study had a number of limitations. For instance, we did not include data on the chemotherapy doses (including dose reductions), G-CSF and EPO utilization, and specific chemotherapy protocols in our analysis. These factors are considered in other toxicity prediction scores, such as the CARG^11^ and the CRASH scores.^10^ The choice of the chemotherapeutic can influence the incidence of toxicities, as certain drugs are associated with specific adverse reactions. It might be useful to incorporate these data into future studies. Lastly, our inclusion of patients having received a GA limits our ability to extrapolate the study’s results to older patients with cancer in general (ie, the target population for whom the question of treatment tolerability is crucial).

## Conclusion

In contrast to severe chemotherapy-related toxicities, moderate toxicities have not been extensively studied and have often been poorly reported in clinical trials. It is essential to consider moderate toxicities (or at least the most relevant ones) before initiating chemotherapy in an older patient. These toxicities can have a significant impact on quality of life and the premature discontinuation of chemotherapy. Here, in a propensity score analysis, we identified anemia for severe toxicities but did not find any for moderate toxicities. Furthermore, after taking into account the decision-making process the type of cancer was not associated with severe toxicities.

## Supplementary material

Supplementary material is available at *The Oncologist* online.

oyae157_suppl_Supplementary_Tables

## Data Availability

Restrictions apply to the availability of these data. Data were obtained from the ELCAPA Study Group and are available from the corresponding author with the permission of the ELCAPA Study Group investigators. The data underlying this article will be shared on reasonable request to the corresponding author.
