# Correction to: Prediction of moderate and severe toxicities of chemotherapy in older patients with cancer: a propensity weighted analysis of ELCAPA cohort

**DOI:** 10.1093/oncolo/oyae225

**Published:** 2024-08-19

This is a correction to: Marc-Antoine Benderra *et al.*, Prediction of moderate and severe toxicities of chemotherapy in older patients with cancer: a propensity weighted analysis of ELCAPA cohort, *The Oncologist*, 2024; oyae157, https://doi.org/10.1093/oncolo/oyae157

In the final sentence of the Results section of the abstract, a typographical correction has been made, updating “Hemoglobin < 0 g/dL” to read “Hemoglobin < 10 g/dL”.

