# Supplementary material for: Prediction of moderate and severe toxicities of chemotherapy in older patients with cancer: a propensity weighted analysis of ELCAPA cohort
Source: Oncologist. 2024 Jul 6;29(11):e1523–31. doi: 10.1093/oncolo/oyae157 (PMC11546720; doi:10.1093/oncolo/oyae157)
Supplement: oyae157_suppl_Supplementary_Tables [file oyae157_suppl_supplementary_tables.pdf]

Table S1. Variables included in the propensity score

Associated with toxicities	Male sex, ECOG-PS >1, CIRS-G >12, Haemoglobin <10 g/dl, Albumin <35 g/L, impaired TUG test time, severe renal failure
Associated with combination chemotherapy	Age <80y, cancer type, metastatic status, ECOG-PS >1, no cognitive impairment

Table S2. Comparison of patient characteristics in the overall cohort before and after OW

	**Before OW**			**After OW**		
	**Single agent chemotherapy (n=293)**	**Combination chemotherapy (n=512)**	**p value[1]**	**Single agent chemotherapy (%)**	**Combination chemotherapy (%)**	**p value[1]**
Age >80y<br>Missing	189 (65)<br>0	274 (54)<br>0	0.002	61	61	1
Female sex<br>Missing	165 (56)<br>0	318 (62)<br>0	0.110	59	59	1
Cancer type						
Digestive	73 (25)	175 (34)	0.006	31	31	1
Gynaecologic	100 (34)	165 (32)	0.580	33	33	1
GU	62 (21)	42 (8)	<0.001	14	14	1
Lung	15 (5)	61 (12)	0.002	8	8	1
Other	43 (15)	68 (14)	0.580	15	15	1
Missing	0	1				
Metastatic status<br>Missing	192 (66)<br>1	270 (53)<br>0	<0.001	60	60	1
ECOG-PS >1<br>Missing	141 (48)<br>3	172 (34)<br>4	<0.001	43	43	1
CIRS-G >12<br>Missing	82 (28)<br>10	129 (25)<br>31	0.390	29	29	1
TUG <20s<br>Missing	197 (67)<br>48	375 (73)<br>74	0.070	80	80	1
Cognitive impairment*<br>Missing	59 (20)<br>16	71 (14)<br>46	0.020	19	19	1
Haemoglobin <10g/dl<br>Missing	42 (14)<br>9	83 (16)<br>18	0.480	16	16	1
Albuminemia <35 g/dl<br>Missing	94 (32)<br>48	160 (31)<br>86	0.810	38	38	1
Creat Cl_{rate} <30 ml/min<br>Missing	18 (6)<br>15	18 (4)<br>37	0.080	5	5	1

The numbers represent N (%) before OW and % after OW
*as judged by the ELCAPA investigator
[1] Kruskall-Wallis and Chi-squared tests for quantitative and qualitative variables respectively, global comparison

Abbreviations: CIRS-G, Cumulative Illness Rating Scale for Geriatrics; Creat Cl_{rate}, Creatine clearance rate; ECOG-PS, Eastern Cooperative Oncology Group Performance Status; GU, genito-urinary; OW, Overlap weighting; TUG, timed up-and-go

Table S3. Associations between patient characteristics and haematological toxicities, without overlap weighting

	**Univariate analysis**						**Multivariate analysis**					
	**Moderate toxicities**			**Severe toxicities**			**Moderate toxicities**		***p***	**Severe toxicities**		***p***
**Variable[&]**	**OR**	**95%CI**	***p***	**OR**	**95%CI**	***p***	**OR**	**95%CI**		**OR**	**95%CI**	
Female sex	0.62	0.45-0.86	0.004	0.67	0.40.97	0.03	0.73	0.42-1.28	0.28	0.95	0.50-1.83	0.89
>80 years of age	1.02	0.74-1.41	0.89	0.94	0.65-1.36	0.74						
Metastatic status	1.42	1.02-1.97	0.04	1.39	0.95-2.03	0.09	0.97	0.62-1.53	0.91	0.88	0.52-1.49	0.64
ECOG-PS>1	1.89	1.36-2.61	<0.001	1.73	1.18-2.52	0.005	1.42	0.88-2.29	0.15	0.97	0.55-1.73	0.92
Polychemotherapy	1.21	0.87-1.71	0.27	1.04	0.71-1.54	0.84						
Haemoglobin <10 g/dl	4.78	2.99-7.63	<0.001	4.49	2.68-7.52	<0.001	6.03	3.00-12.10	<0.001	5.47	2.53-11.81	<0.001
Creatine clearance rate <30 ml/min	1.21	0.52-2.81	0.66	2.70	1.25-5.82	0.01	1.07	0.37-3.15	0.90	2.25	0.79-6.42	0.13
Albuminemia<35 g/L	1.99	1.39-2.84	<0.001	2.19	1.45-3.32	<0.001	1.17	0.72-1.91	0.53	1.42	0.80-2.52	0.23
6-month weight loss	1.35	0.92-1.98	0.13	1.23	0.78-1.93	0.37	1.01	0.61-1.69	0.96	1.04	0.57-1.90	0.89
CIRS-G >12	1.36	0.95-1.95	0.09	1.30	0.86-1.98	0.22	0.98	0.61-1.59	0.94	0.92	0.52-1.63	0.77
ADL <6	1.28	0.80-2.04	0.31	1.18	0.68-2.05	0.55						
IADL <7	1.30	0.94-1.81	0.12	1.34	0.92-1.95	0.13						
TUG <20 s	0.72	0.46-1.14	0.16	0.79	0.46-1.37	0.41	1.03	0.63-1.68	0.92	0.79	0.43-1.45	0.45
Cognitive impairment	0.90	0.57-1.41	0.64	1.14	0.70-1.85	0.59						
BMI<br><22 vs 22-25 kg/m²<br>>25 vs 22-25 kg/m²	 1.21<br>1.01	 0.79-1.86<br>0.68-1.50	 0.37<br>0.95	 0.94<br>0.81	 0.58-1.53<br>0.52-1.27	 0.81<br>0.35						
Gynaecological cancer*	0.70	0.47-1.06	0.09	0.78	0.48-1.26	0.31	1.21	0.64-2.27	0.55	1.15	0.55-2.41	0.70
GU cancer*	1.23	0.73-2.06	0.44	1.63	0.91-2.91	0.10	0.98	0.47-2.05	0.95	1.76	0.78-3.99	0.18
Lung cancer*	1.68	0.96-2.93	0.07	1.46	0.74-2.89	0.27	1.96	0.94-4.09	0.07	1.51	0.58-3.90	0.40
Other cancer*	0.67	0.39-1.15	0.15	1.04	0.58-1.86	0.91	0.59	0.27-1.28	0.18	1.04	0.46-2.37	0.92

[&]For continuous variables, the Youden Index30 was used to identify the cut-off with the highest sensitivity and specificity for classifying the presence or absence of toxicity. Variables with p<.1 in the univariate analysis and clinically relevant variables (single-agent chemotherapy or combination chemotherapy) were examined further in a multivariate logistic regression model.

*reference: digestive cancer

Abbreviations: ADL, Activities of Daily Living; BMI, body mass index; CIRS-G, Cumulative Illness Rating Scale for Geriatrics; ECOG-PS, Eastern Cooperative Oncology Group Performance Status; GU, genito-urinary; IADL, Instrumental Activities of Daily Living; TUG, timed up-and-go

Table S4. Association between patient characteristics and non-haematological toxicities, without overlap weighting

	Univariate analysis						Multivariate analysis					
	Moderate toxicities			Severe toxicities			Moderate toxicities		*p*	Severe toxicities		*p*
**Variable[&]**	**OR**	**95%CI**	***p***	**OR**	**95%CI**	***p***	**OR**	**95%CI**		**OR**	**95%CI**	
Female sex	1.20	0.88-1.63	0.25	0.90	0.60-1.33	0.58						
>80 years of age	0.90	0.67-1.22	0.50	1.34	0.90-2.01	0.15	0.74	0.52-1.06	0.10	1.34	0.84-2.13	0.22
Metastatic status	0.99	0.73-1.34	0.94	1.34	0.90-2.01	0.15	0.94	0.66-1.35	0.75	1.03	0.65-1.65	0.89
ECOG-PS>1	1.22	0.90-1.66	0.21	1.33	0.89-1.99	0.16	1.44	0.98-2.11	0.06	1.52	0.94-2.47	0.09
Polychemotherapy	1.42	1.03-1.96	0.03	0.89	0.59-1.35	0.59	1.35	0.93-1.96	0.12	1.00	0.63-1.61	0.98
Haemoglobin <10 g/dl	0.92	0.60-1.41	0.71	1.07	0.63-1.81	0.81						
Creatine clearance rate <30 ml/min	1.24	0.61-2.51	0.55	0.68	0.23-2.05	0.50						
Albuminemia <35 g/L	1.09	0.78-1.54	0.60	1.30	0.84-2.00	0.24						
6-month weight loss	1.12	0.78-1.61	0.55	1.03	0.64-1.67	0.89						
CIRS-G >12	0.97	0.68-1.37	0.85	1.21	0.79-1.86	0.39						
ADL <6	1.32	0.85-2.05	0.22	0.88	0.47-1.64	0.68						
IADL <7	1.16	0.85-1.57	0.36	1.07	0.71-1.60	0.76						
TUG <20 s	0.73	0.48-1.13	0.16	0.98	0.55-1.75	0.94	0.77	0.48-1.24	0.29	1.21	0.63-2.31	0.57
Cognitive impairment	1.02	0.67-1.55	0.93	1.38	0.83-2.28	0.21						
BMI												
<22 vs 22-25 kg/m²	0.82	0.55-1.22	0.33	0.88	0.51-1.51	0.63	0.88	0.56-1.40	0.60	0.87	0.46-1.65	0.67
>25 vs 22-25 kg/m²	1.14	0.79-1.64	0.48	1.49	0.93-2.40	0.10	1.30	0.86-1.96	0.22	1.83	1.07-3.14	0.03
Gynaecological cancer*	0.90	0.62-1.30	0.57	0.63	0.38-1.05	0.07	0.92	0.60-1.41	0.71	0.56	0.32-0.99	0.05
GU cancer*	0.76	0.45-1.27	0.29	1.35	0.76-2.39	0.31	0.82	0.45-1.49	0.51	1.13	0.57-2.23	0.73
Lung cancer*	0.61	0.34-1.08	0.09	0.79	0.40-1.57	0.50	0.55	0.28-1.10	0.09	0.73	0.32-1.65	0.45
Other cancer*	0.93	0.58-1.49	0.75	0.53	0.26-1.07	0.08	0.83	0.46-1.48	0.52	0.52	0.24-1.17	0.11

[&]For continuous variables, the Youden Index30 was used to identify the cut-off with the highest sensitivity and specificity for classifying the presence or absence of toxicity. Variables with p<.1 in the univariate analysis and clinically relevant variables (single-agent chemotherapy or combination chemotherapy) were examined further in a multivariate logistic regression model.

*reference: digestive cancer

Abbreviations: ADL, Activities of Daily Living; BMI, body mass index; CIRS-G, Cumulative Illness Rating Scale for Geriatrics; ECOG-PS, Eastern Cooperative Oncology Group Performance Status; GU, genito-urinary; IADL, Instrumental Activities of Daily Living; TUG, timed up-and-go
